# Exploring the effects of demonstration and enactment in facilitating recall of instructions in working memory

**DOI:** 10.3758/s13421-019-00978-6

**Published:** 2019-11-25

**Authors:** Richard J. Allen, Liam J. B. Hill, Lucy H. Eddy, Amanda H. Waterman

**Affiliations:** 1 School of Psychology, University of Leeds, Leeds, West Yorkshire LS2 9JT UK; 2 Bradford Institute for Health Research, Bradford Teaching Hospitals Foundation Trust, Bradford, West Yorkshire UK

**Keywords:** Memory, Motor control, Recall, Sequence learning, Working memory

## Abstract

Across the lifespan the ability to follow instructions is essential for the successful completion of a multitude of daily activities. This ability will often rely on the storage and processing of information in working memory, and previous research in this domain has found that self-enactment at encoding or observing other-enactment at encoding (demonstration) improves performance at recall. However, no working memory research has directly compared these manipulations. Experiment 1 explored the effects of both self-enactment and demonstration on young adults’ (N=48) recall of action-object instruction sequences (e.g. ‘spin the circle, tap the square’). Both manipulations improved recall, with demonstration providing relatively larger boosts to performance across conditions. More detailed analyses suggested that this improvement was driven by improving the representations of actions, rather than objects, in these action-object sequences. Experiment 2 (N=24) explored this further, removing the objects from the physical environment and comparing partial demonstration (i.e. action-only or object-only) with no or full demonstration. The results showed that partial demonstration only benefitted features that were demonstrated, while full demonstration improved memory for actions, objects and their pairings. Overall these experiments indicate how self-enactment, and particularly demonstration, can benefit verbal recall of instruction sequences through the engagement of visuo-motor processes that provide additional forms of coding to support working memory performance.

**Electronic supplementary material:**

The online version of this article (10.3758/s13421-019-00978-6) contains supplementary material, which is available to authorized users.

## Introduction

Across the lifespan the ability to follow instructions is crucial to success in everyday life. It plays a central role in learning new skills, is important for a multitude of daily activities such as cooking and technology use, and has been identified as a key activity underpinning learning within the classroom (Gathercole, Durling, Evans, Jeffcock, & Stone, 2007; Gathercole, Pickering, Knight, & Stegmann, 2003). The ability to remember and implement instructions is often thought to rely on the storage and processing of information in working memory (Gathercole et al., 2007; Waterman et al., 2017; Yang, Allen, & Gathercole, 2016a); a limited capacity system for the temporary maintenance and processing of information being used in ongoing cognition and action (Baddeley, 2012; Cowan, 2012; Logie, 2011). Thus, working memory tasks typically require immediate recall or recognition of highly constrained sets of information, in line with the generally accepted limited capacity (around four items) and retention duration (approximately a few seconds, using filled delays) of the working memory system (see Cowan, 2010; Oberauer et al., 2018, for reviews). When using list recall, serial order is also usually required as a feature of the task. The central role of working memory in broader cognition in general, and in following instructions specifically, means it is useful to identify and understand methods of improving working memory for instructions, both from a theoretical and a practical perspective. The current study was therefore designed to explore two manipulations (self-enactment and demonstration), that can be applied during the initial encoding of instructions, to better understand how these manipulations impact following instruction (FI) performance in working memory tasks.

An established literature exists in the long-term memory (LTM) domain indicating that self-enactment by the participant during encoding (often termed the ‘subject-performed task’ (SPT) effect) and/or visually demonstrating instructions to the participant (the ‘experimenter-performed task’ (EPT) or observation effect) can influence and often benefit later memory performance (e.g. Bäckman & Nilsson, 1984; Badinlou, Kormi-Nouri, & Knopf, 2017, 2018; Cohen, 1989; Cohen & Bean, 1983; Engelkamp, 1998; Engelkamp & Dehn, 2000; Engelkamp & Zimmer, 1989; Kormi-Nouri, 1995, 2000; Schult, von Stülpnagel, & Steffens, 2014; Steffens, 2007; Steffens, Jelenec, & Mecklenbräuker, 2009; Steffens, von Stülpnagel, & Schult, 2015; von Stülpnagel, Schult, Richter, & Steffens, 2016). This relatively extensive existing literature has yielded a number of insights concerning how each of these encoding-based manipulations might impact on ‘action memory’ across different populations, paradigms, and methodological contexts. However, these studies have generally explored memory for extended lists of items, often over lengthy retention intervals or shorter intervals, but with participants required to perform filler tasks during this interval that serve to pre-occupy working memory capacity. They often also control for recency effects, associated with working memory storage, and thus these types of studies use methodologies that purposefully exceed working memory capacity and instead primarily emphasize investigation of LTM function. In sum, while such research might indirectly have implications for the interpretation of outcomes derived from working memory, it is highly likely that a range of different mechanisms are in operation during short-term/working memory and long-term memory tasks (e.g. Baddeley, 1966a, 1966b; Cowan, 2008; Norris, 2017). It is therefore important to view the aforementioned existing literature as at least somewhat distinct, and to focus instead on exploring how enactment and demonstration might impact on working memory specifically.

In one of the first studies to do so, Allen and Waterman (2015) presented sequences of verbal instructions (e.g. *Spin the triangle, tap the circle,* etc.) and asked participants to perform (‘enact’) each of these action-object pairings on shapes laid out in front of them, as they were presented during encoding. They found that self-enactment during encoding particularly benefited subsequent verbal recall of the sequence, with little impact on enacted recall. This observation of positive encoding-enactment effects, which are particularly prevalent with verbal (rather than enacted) recall, has also been observed in children (Jaroslawska, Gathercole, Allen, & Holmes, 2016; Waterman et al., 2017, Experiment 3). One suggested mechanism for this finding is that self-enactment at encoding encourages non-strategic, automatic engagement with additional visuo-spatial and motoric codes that facilitate verbal recall (Waterman et al., 2017). This is consistent with the reduced influence of self-enactment at encoding on enacted recall: where participants are already engaging with the additional forms of visuospatial-motoric coding because they are planning for physical performance of the instructions later (Allen & Waterman, 2015; Jaroslawska, Gathercole, & Holmes, 2018). Further support for this idea comes from Jaroslawska et al. (2016) who found that simply repeating the verbal instructions orally at encoding (instead of enacting the verbal instructions at encoding) did not improve recall.

Demonstration has also recently been explored in the context of working memory. Waterman et al. (2017) found that having the experimenter provide visual demonstration alongside verbal presentation benefited children’s memory for instructions (see also Wojcik, Allen, Brown, & Souchay, 2011) to an equivalent extent for verbal and enacted recall. Yang, Allen, Yu, and Chan (2016b) extended this to the use of video demonstration (see also Yang, Jia, Zheng, Allen, & Ye, 2018) finding that this facilitated adults’ recall performance, compared to auditory presentation of verbal instruction sequences. This was further observed in both children with attention-deficit hyperactivity disorder (ADHD) and age-matched typical controls (Yang, Allen, Holmes, & Chan, 2017). Thus, it is apparent that visual demonstration can benefit FI performance in working memory. Waterman et al. (2017) suggest that the benefits derived from demonstration at encoding may operate in a similar way to those derived from self-enactment at encoding. Demonstration also provides additional visuospatial-motoric representations via observation of another’s enactment. This is consistent with research showing a shared neuronal substrate for motoric performance and observation of motoric acts (Hickok & Poeppel, 2004; Nelissen, Luppino, Vanduffel, Rizzolatti, & Orban, 2005; Rizzolatti & Luppino, 2001).

However, whilst the impacts of encoding-based enactment and demonstration have been observed in different tasks and populations (Allen & Waterman, 2015; Jaroslawska et al., 2016, 2018; Waterman et al., 2017) and have been proposed as mechanistically similar, no working memory study has yet directly compared both manipulations in a single experiment. The closest existing example is a set of studies by Helstrup (2001, 2005), who examined immediate serial recall of simple sequences of self-referent actions (moving one’s hand or body through a series of locations within a 5 × 5 grid).

They consistently found benefits from demonstration but not self-enactment in this task. However, these studies tested sequence lengths of up to ten items, which would conceivably involve recruitment of both working and long-term memory. This study also employed a paradigm that assessed memory for sequences of simple movements around a constrained grid of locations, and so primarily represents a measure of spatial memory. In contrast, the present study, and the growing field of research exploring working memory for instructions, focuses on memory for more complex sequences involving multiple distinct actions and objects, and thus emphasize visual and motoric processing while rendering spatial location an implicit aspect of the task.

The absence of work directly comparing self-enactment and demonstration limits our understanding of the extent to which these manipulations genuinely represent overlapping or distinct forms of processing. Directly comparing demonstration and self-enactment is also important given Waterman et al.’s (2017) observation that, in young children, the benefits of self-enactment but not demonstration at encoding were dependent on sequence complexity (i.e. sequence length and number of possible actions and objects in the experimental set). Demonstration boosted recall regardless of level of complexity, whereas self-enactment benefits were only evident when sequences were simplified (i.e. shorter sequences with less variation in the possible actions or objects involved). Waterman et al. (2017) suggest this may reflect the increased cost associated with self-enactment compared to demonstration. In other words, self-enactment is potentially a more resource-intensive, attentionally demanding means of acquiring information compared to passive observation. Any benefits from self-enactment are therefore counterbalanced by the competing attentional costs of self-generating the visuospatial-motoric information, a cost paid on ones’ behalf by the experimenter when simply observing.

With there findings in mind it is possible demonstration, rather than self-enactment, offers a preferable means of enhancing encoding because it takes advantage of the same additional storage mechanisms but at a lower cost to cognitive resources. The present study was therefore designed to further our understanding of whether encoding-based self-enactment had additive benefits over and above observation of other-enactment (i.e. demonstration). In other words, does self-enactment have any benefits to offer in addition to demonstration or do both these mechanisms operate via enhancing encoding of visuo-spatial and motoric aspects of a sequence and thus are functionally equivalent? Experiment 1 examined verbal recall of instruction sequences following either verbal presentation or visual demonstration and manipulated whether or not participants also enacted each element of the sequence during encoding. In response to questions raised by the results of Experiment 1, Experiment 2 then further explored *how* demonstration benefits memory for instructions, by systematically manipulating the degree of information provided at encoding via demonstration and examining resulting impacts across different FI performance measures.

## Experiment 1

Experiment 1 directly compared verbal recall of instructions following either auditory-verbal or visually demonstrated presentation and examined whether self-enactment of instructions during encoding had an additive benefit in each of these conditions. Previous research has established that self-enactment leads to superior recall accuracy relative to verbal presentation, with this attributed to the beneficial engagement of visuospatial-motoric coding (Allen & Waterman, 2015; Jaroslawska et al., 2016). Similarly, use of this additional coding has also been hypothesized to underlie the positive effects of demonstration that have previously been observed (Waterman et al., 2017; Yang, Allen, & Gathercole, 2016a). Therefore, if self-enactment and demonstration effects have similar underlying mechanisms, we would predict them to have non-additive impacts on performance (akin to those observed between enactment at encoding and enactment at recall, e.g. Allen & Waterman, 2015; Jaroslawska et al., 2016). In addition, Waterman et al. (2017) have suggested that demonstration provides these additional codes at reduced cost compared to self-enactment. This study therefore also provides the opportunity to investigate if there are differences in the magnitude of boosts to recall between self-enactment and demonstration.

Previous working memory FI research has used both everyday objects such as stationery items (e.g. Charlesworth, Allen, Morson, Burn, & Souchay, 2014; Jaroslawska et al., 2016; Yang, Allen, Yu, et al., 2016; Yang, Gathercole, & Allen, 2014; Yang et al., 2018) or geometric shapes (e.g. Allen & Waterman, 2015; Waterman et al., 2017). The current study used geometric shapes and interchangeable novel action-object pairings to help ensure participants could not draw on pre-existing associations between elements of a sequence, from LTM, to aid recall (Allen & Waterman, 2015). Pre-recorded audio and video clips were also used to present the sequences to be learned (as in Yang, Allen, Yu, et al., 2016; Yang et al., 2018) to maximize standardization of procedures.

The effects of presentation type and self-enactment during encoding were explored using recall of action-object pairs as the initial outcome variable (Allen & Waterman, 2015; Charlesworth et al., 2014; Jaroslawska et al., 2016, 2018; Waterman et al., 2017; as in Wojcik et al., 2011; Yang, Allen, & Gathercole, 2016a; Yang et al., 2014, 2018). When examining precisely how these factors might affect different aspects of performance, it is also useful to break down recalled instructions into their constituent parts. Therefore, we included independent recall of actions and objects as additional outcome variables.

### Method

#### Participants

The sample consisted of 48 young adults (22 male and 26 female) with a mean age of 22.65 years (*SD*= 3.14 years), acquired using the University of Leeds Participant Pool Scheme. Participants were native English speakers with normal or corrected-to-normal vision and with no history of neurological disorders. Ethical approval was granted by the University of Leeds School of Psychology Research Ethics Committee (reference number: 16-0339).

#### Design and procedure

The experiment used a 2 × 2 repeated-measures design, manipulating the use of demonstration at encoding (presentation type) and the use of self-enactment at encoding (enactment). Instructions were either presented verbally or were visually demonstrated, and the participant either acted out the instructions at encoding (self-enactment) or simply listened to/watched the instruction sequences at encoding (no-enactment), creating four experimental conditions presented in a counterbalanced order. The verbal conditions required participants to listen to instructions through a pair of headphones, played from a desktop computer. In the demonstration conditionsparticipants watched a silent video of a hand carrying out the instructions on a set of objects (as in Yang, Allen, & Gathercole, 2016a). For the self-enactment conditionsparticipants performed the action-object pairs on objects laid out in front of them.

Consistent with previous research (Allen & Waterman, 2015; Waterman et al., 2017) all instructions comprised a sequence of actions (e.g. *spin*) and objects (e.g. *triangle*), which together formed action-object pairs (e.g. *spin the triangle*). For each condition, participants had four practice trials (two consisting of three action-object pairs, and two with four pairs). Experimental trials for each condition included six trials with sequences of four action-object pairs, followed by six trials with sequences of five pairs, in order to allow for variation in working memory ability across individuals.

After completing each instruction sequence a cue (the text *‘Recall Now’*) appeared on the screen, this was 3 s after the presentation of the last instruction. Participants attempted to verbally recall each sequence of instructions in their originally presented serial order. For all conditions, the pre-task instructions were presented on-screen and simultaneously read aloud by the experimenter, to ensure understanding of the requirements of each task. Prior to practice trials there was also a familiarization period before testing began to ensure that participants could name all objects and actions and replicate the instructions physically.

#### Materials

Six shapes (*circle, cross, square, star, sun and triangle*) and six actions (*drag, flip, lift, push, spin and touch*) were used in this experiment. Shapes and actions were repeatedly recombined to form the action-object pairs that constituted each sequence of items (such that, for example, *square* might be paired with *drag* on one trial but with *spin* on the next), with the constraint that no action or object repeated within a given sequence. The shapes were presented as solid black shapes on a white background, measuring 5 × 5 cm. These were mounted, double sided, on cork coasters that were cut to size, to make the shapes easier to manipulate. For all conditions, these shapes were present in front of the participant, in the same configuration as they appeared in the videos.

**Fig. 1:**
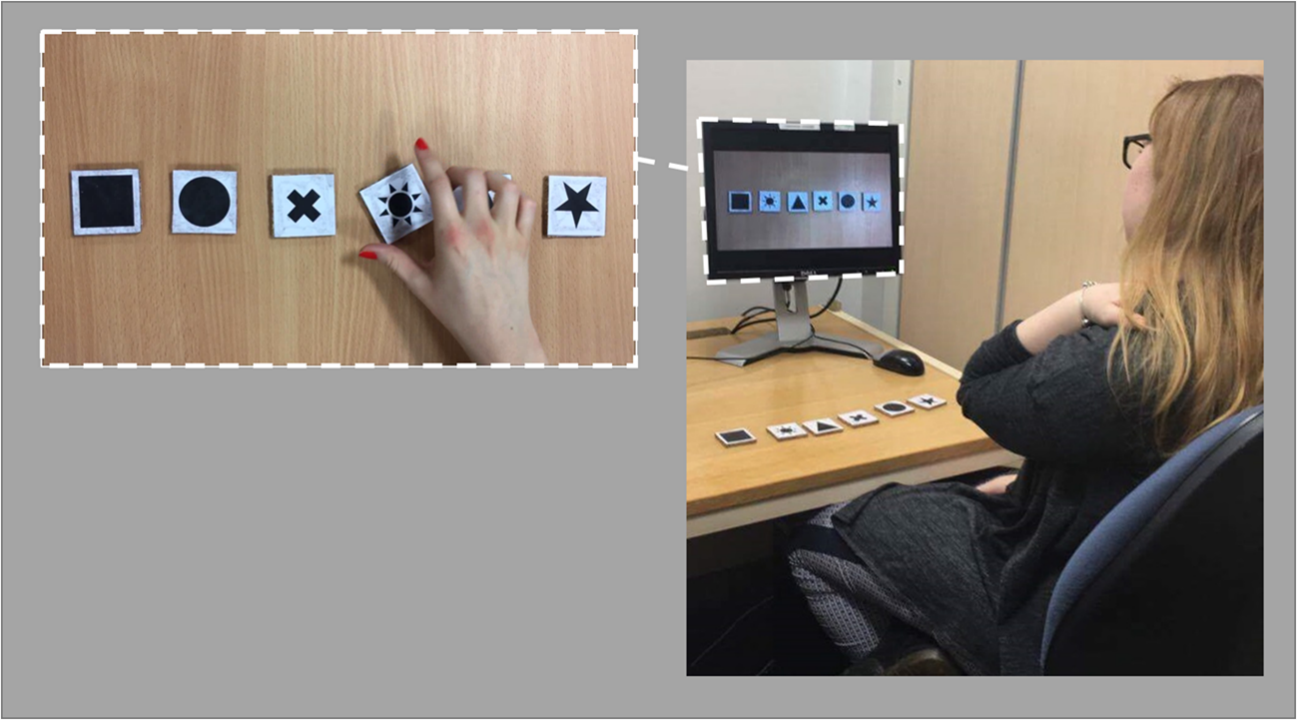
Illustration of the experimental setup. *Note:* In conditions involving verbal instruction the monitor screen was blank. For demonstration conditions participants viewed an on-screen hand act out the sequence, as illustrated in the pop-out image (showing the object-action ‘*spin the sun*’)

In order to present the instructional stimuli on a computer, the experiment was built in PsychoPy (http://www.psychopy.org/). Within the demonstration conditions silent videos of a sequence of demonstrated instructions were presented, these had the shapes placed 3 cm apart on a plain table (see Fig. 1). When a shape was pushed or dragged it would move 10 cm from its starting location and was then returned to its starting position. A hand was shown demonstrating each action-object pair for approximately 3 s, followed by a 3-s break until the commencement of the next pair (to allow time for self-enactment in the relevant conditions). Within these conditions shapes remained visible on-screen throughout the encoding phase of each trial. Within the verbal conditions, audio-only clips of the action-object pairs within a sequence were presented. As with the demonstration conditions, presentation of each action-object pair was separated by a 3-s interval. The computer screen remained blank throughout the verbal conditions.

#### Data analysis

Appropriate frequentist statistical techniques were employed in both Experiment 1 and Experiment 2. However, due to the fact that these tests rely on null hypothesis significance testing, they cannot provide evidence of no effect, only a non-significant result (Barchard, 2015). For this reason Bayes factor analyses were conducted alongside frequentist statistics using JASP (https://jasp-stats.org/). Bayes factor analyses allow a direct comparison between the alternative hypothesis and the null hypothesis (Mulder & Wagenmakers, 2016; Wagenmakers et al., 2018). The results of the Bayes factor and frequentist analyses are presented together.

Consistent with previous work (e.g. Allen & Waterman, 2015; Jaroslawska et al., 2016; Waterman et al., 2017), mean proportion of action-object pairs correctly recalled in the correct serial order was adopted as the primary dependent variable. To provide further insight into how recall is influenced by encoding context performance was also broken down into recall of actions and of objects, independently.

### Results

**Fig. 2:**
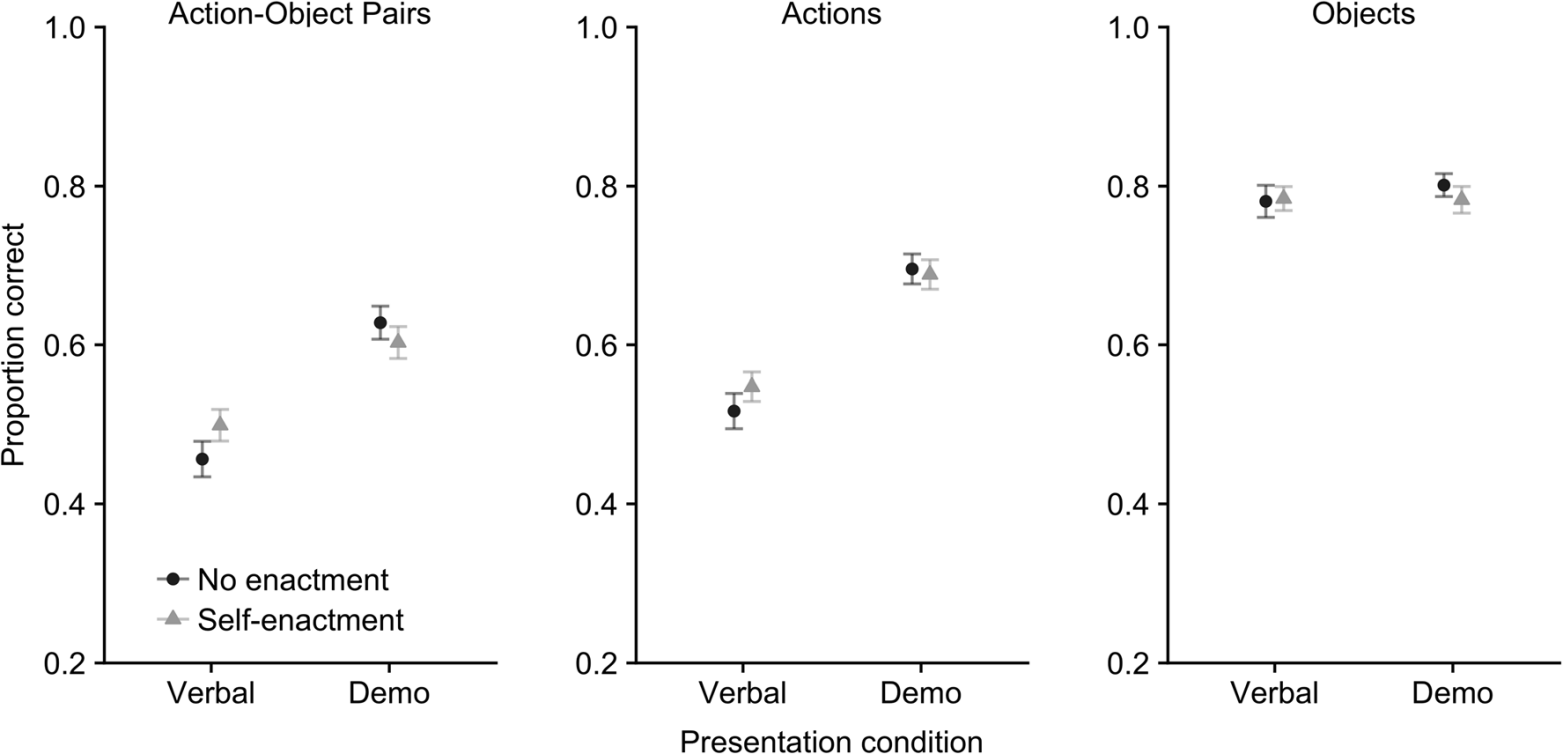
Mean proportion recalled by presentation and enactment condition for Action-Object pairs, Actions-only and Objects-only outcome measures (with standard error)

Descriptive statistics for performance on the following instructions task are presented in Fig. 2 for all three outcome measures.

#### Action-object pairs

A 2 (presentation type: demonstration vs. verbal) × 2 (enactment: self-enactment vs. no-enactment) repeated-measures ANOVA indicated a significant main effect of presentation type *F*(1,47) = 85.33, MSE = .01, *p*<.001, *η*^*2*^*p* =.65, *BF*_*10*_ = 1.38E+13, whereby demonstration leads to greater recall accuracy (*M* = .62, *SE* = .02) than verbal instruction (*M* = .48, *SE* = .02). No significant main effect of enactment was found *F*(1,47) =.38, MSE = .01, *p* =.54, *η*^*2*^*p* =.01, *BF*_*10*_ = .19, but there was a significant interaction between presentation type and enactment *F*(1,47) = 5.17, MSE = .01, *p* =.028, *η*^*2*^*p* =.10, *BF*_*10*_ = 2.17. The Bayesian analysis indicated strongest support for the model containing presentation type and the presentation × enactment interaction (*BF*_*10*_*=* 5.23E+13 compared to the null model, and 2.24 more likely than the next best model containing presentation type only).

Further comparisons were carried out to unpack the interaction. These indicated significantly increased recall accuracy in the demonstration condition, relative to verbal presentation, for both no-enactment, *t*(47) = 7.39, *p* < .001, *d* = 1.01, *BF*_*10*_ = 5.524E+6, and self-enactment, *t*(47) = 5.56, *p* < .001, *d* = .80, *BF*_*10*_ = 13491. With regard to self-enactment, this positively impacted on performance within the verbal presentation condition, *t*(47) = 2.08, *p* = .043, *d* = .30, *BF*_*10*_ = 1.12, but not the demonstration condition, *t*(47) = 1.19, *p* = .24, *d* = .17, *BF*_*10*_ = .31 (although it should be noted that the Bayes factor support for the positive impact of self-enactment in the verbal condition was ambiguous and therefore should be treated with caution).

#### Actions

A 2 × 2 repeated-measures ANOVA indicated a significant main effect of presentation type *F*(1,47) = 143.25, MSE = .01, *p* < .001, *η*^*2*^*p* =.75, *BF*_*10*_ = 4.541E+19, whereby demonstration led to greater recall accuracy (*M* = .70) than verbal instruction (*M* = .54). There was no significant main effect of enactment, *F*(1,47)=.78, MSE = .01, *p*=.38, *η*^*2*^*p*=.02, *BF*_*10*_ = .22, and no interaction between presentation type and enactment *F*(1,47)=1.81, MSE = .01, *p*=.18, *η*^*2*^*p*=.04, *BF*_*10*_ = .50. The Bayesian analysis indicated strongest support for the model containing just presentation type (*BF*_*10*_ = 4.44E+19 compared to the null model, and 4.43 more likely than the next best model containing presentation type and enactment).

#### Objects

A 2 × 2 repeated-measures ANOVA indicated no significant main effect of presentation type *F*(1,47) = .64, MSE = .01, *p* =.43, *η*^*2*^*p* =.01, *BF*_*10*_ = .194, enactment, *F*(1,47) =.31, MSE = .01, *p* = .58, *η*^*2*^*p* = .01, *BF*_*10*_ = .19, or the interaction between presentation type and enactment *F*(1,47) = .70, MSE = .01, *p* =.41, *η*^*2*^*p* =.02, *BF*_*10*_ = .29. The Bayesian analysis indicated strongest support for the null model (at least 5.15 times more likely than any other model).

### Discussion

Recall of action-object pairs was superior following demonstration of instructions, relative to verbal presentation, replicating the findings of previous working memory studies (Waterman et al., 2017; Yang, Allen, & Gathercole, 2016a; Yang et al., 2017). A positive effect of self-enactment during encoding was also found, though this was qualified by an interaction with presentation type and was only observed in the verbal presentation condition. Self-enactment did not provide any additional boost to recall in the demonstration condition. This pattern is broadly analogous to those previously observed when combining enactment at encoding and enactment at recall (Allen & Waterman, 2015; Jaroslawska et al., 2016; Waterman et al., 2017).

It supports the hypothesis that self-enactment and demonstration encourage engagement with overlapping forms of representational coding. In other words, both demonstration at encoding and self-enactment at encoding provide similar, additional, forms of visuospatial-motoric coding that supplement verbal codes in working memory. Therefore, the effect of these two manipulations is non-additive and providing additional codes through *only one* of these manipulations is sufficient to boost verbal recall.

However, the self-enactment effect was weak, even in the verbal condition, with a relatively small effect and inconclusive Bayes factor support. This supports the suggestion that demonstration at encoding is a more robust way to boost recall (Waterman et al., 2017).

These patterns change when considering action and object separately though. For actions demonstration led to improved verbal recall. Meanwhile self-enactment did not improve recall accuracy, even in the verbal presentation condition. When considering objects, neither self-enactment nor demonstration boosted recall accuracy. On the face of it this appears to suggest that demonstration primarily operates via improving representations of actions in object-action sequences. However, before considering that idea further it is important to note that actions, in contrast to objects, are not present in the environment unless they are demonstrated or self-enacted. In Experiment 1 objects were always present (even in the verbal condition), 1, to enable the participant to perform the sequences in the self-enactment conditions. Indeed, all previous studies investigating FI in working memory have used a methodology where the objects are always present during encoding. This means that participants have the opportunity to visually engage with the objects, but not actions, even when simply listening to verbal instructions. This opportunity for the passive cueing of objects at the encoding stage may not leave room for any further improvements to occur in object recall via active demonstration.

Experiment 2 therefore investigated whether or not additional visuospatial-motoric codes could improve recall of objects when the objects were not visible during encoding. This necessitated a focus on demonstration, rather than self-enactment, because of the difficulties of engaging in self-enactment in the absence of physical objects. Furthermore, Experiment 2 was also designed to explore in more detail *how* demonstration aids verbal recall. Indeed, it would be interesting to clarify what aspects of the visual information provided by observation are particularly important in aiding memorization. Demonstration of object-action sequences obviously provides information on both objects and actions, through observing the appropriate action being used on the relevant object. What might happen though if only partial information is provided via demonstration: action only or object only? Will this provide a partial boost to verbal recall (in being less effective than full demonstration, but more effective then no demonstration)? Alternatively, do both object and action have to be cued in order to improve verbal recall over and above a condition without any demonstration?

## Experiment 2

Previous studies in this area (e.g. Waterman et al., 2017; Yang, Allen, & Gathercole, 2016a; Yang et al., 2017) have always displayed objects in front of the participants during the encoding phase.

This has meant that object information, unlike action information, is always available during encoding. Experiment 1 found that demonstration had a robust, positive effect on memory for action-object pairs, and for the individual actions but not for individual object memory. However, object-availability at encoding may reduce the opportunity for manipulations to reinforce object information (via demonstration). The current experiment explored this idea by removing the presence of the physical objects at encoding and looking at whether this changed the impact of demonstration on object accuracy.

In addition, Experiment 2 looked at how partial demonstration would impact accuracy across the different outcome variables by comparing performance on a full demonstration condition with a verbal-only condition and also two ‘partial’ demonstration conditions involving either only actions or only objects. Two straightforward predictions would be that demonstrating a single-feature dimension would aid memory for that feature, and that full demonstration will lead to superior recall performance compared with a verbal only presentation. An interesting further question is whether partial demonstration, of either the action only or the object only, boosts memory for the other feature, relative to the no-demonstration (verbal-only) condition. In other words, do the benefits of demonstrating *either* aspect of an action-object pairing generalize to all aspects of that to-be-recalled pair or do *both* features need to be demonstrated to provide a significant boost to recall?

In order to explore these questions a few methodological changes were necessary compared to Experiment 1. Firstly, verbal presentation of instruction sequences was always present in all conditions. This approach mirrors the form of demonstration context used in previous working memory studies (e.g. Waterman et al., 2017) and was necessary given the inclusion of partial demonstration conditions in this experiment. Secondly, as previously discussed, the physical objects were not present in the current experiment.

### Method

#### Participants

The sample comprised 24 young adults: nine males and 15 females, with a mean age of 27.5 years (*SD*= 5.2 years). The sample was acquired using the University of Leeds Participant Pool Scheme. No participants who had taken part in Experiment 1 were part of the current sample. All participants were native English speakers, with normal or corrected-to-normal vision and with no history of neurological disorders. Participants were paid £6 for their time. This research was granted ethical approval by the University of Leeds School of Psychology Research Ethics Committee (reference number: 17-0206).

#### Design

This experiment used a repeated-measures design, in which participants completed all four conditions (no demonstration; action-only demonstration; object-only demonstration; full demonstration) in a counterbalanced order. Performance was measured using the mean proportion correct of pairs correctly recalled as the initial dependent variable, with action-only recall and object-only recall as additional outcome variables. Responses were scored as correct when recalled in the correct sequence position.

#### Procedure and materials

**Fig. 3:**
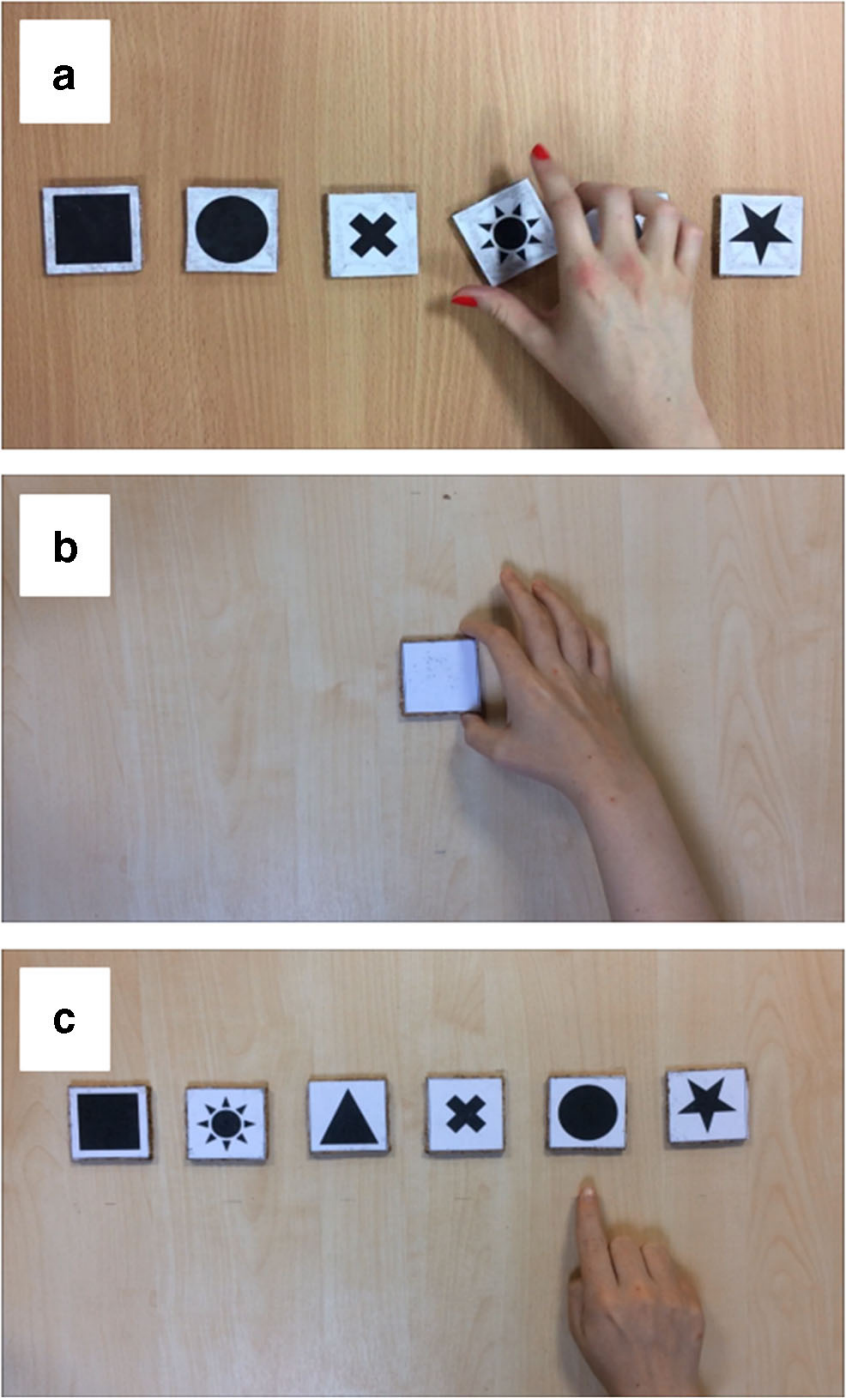
Stills depicting the three forms of demonstration used in Experiment 2: (**A**) Full, (**B**) Action only and (**C**) Object only

The materials used were the same as those used in Experiment 1 except for the following changes: (1) The objects were not present on the table in front of the participant during encoding (or at any other point); and (2) all videos included voice recordings of the action-object sequences. In the action-only demonstration condition a neutral blank shape was used. This blank shape comprised a 5 × 5 cm cork square with plain white paper mounted on both sides. For the object-only demonstration condition, the video showed a hand pointing (3 cm away from the shape) towards the object heard in the voice recording (see Fig. 3). Participants now did not have to engage in self-enactment for any of the conditions in this experiment, instead they simply had to listen to the voice recording and watch the laptop screen (which was blank for the no-demonstration condition) during encoding. The procedure for recall was the same as Experiment 1.

### Results

**Fig. 4:**
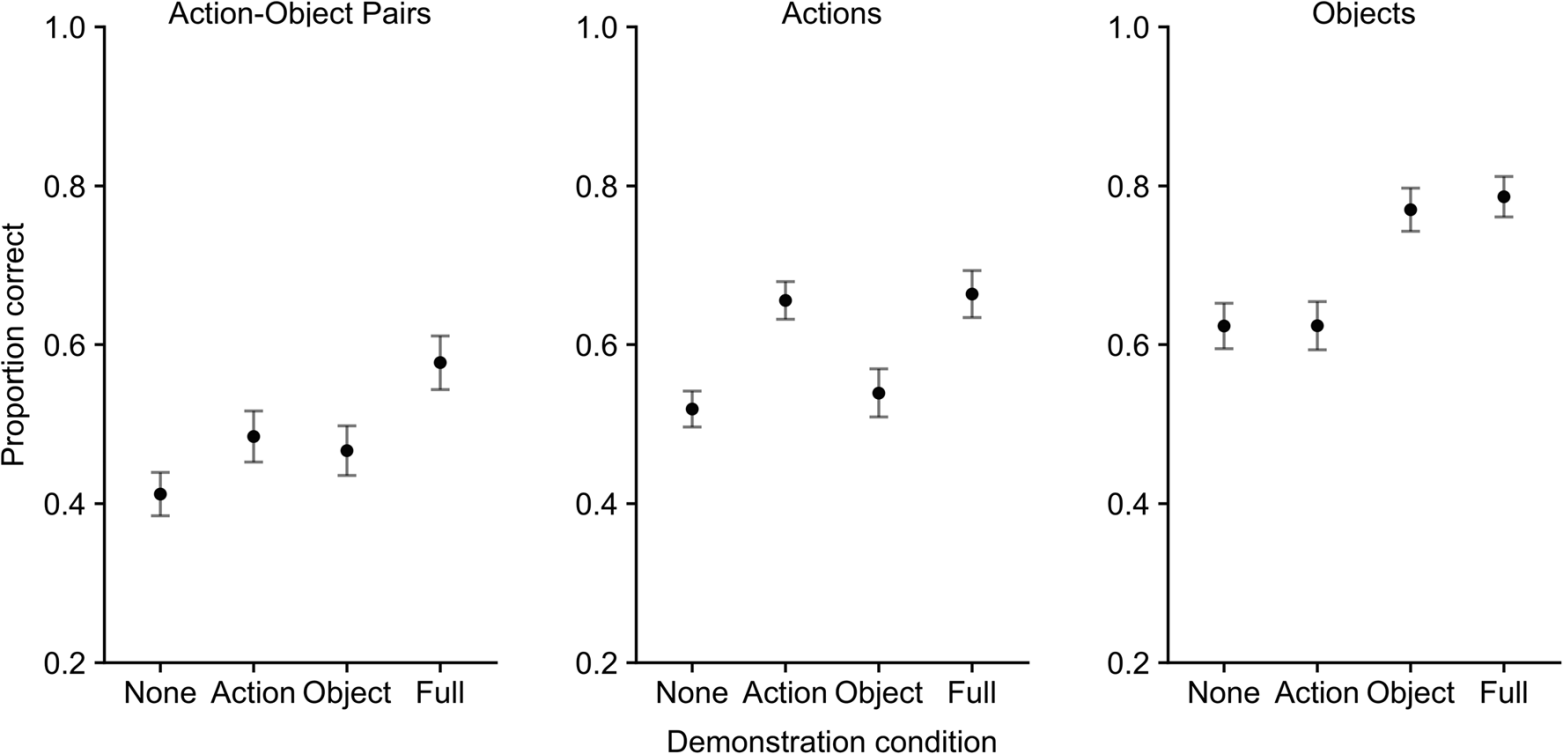
Mean proportion recalled by condition for Action-Object pairs, Actions-only and Objects-only outcome measures (with standard error)

Descriptive statistics for each outcome measure are presented in Fig. 4.

#### Action-object pairs

Frequentist and Bayesian one-way ANOVA were carried out on the proportion of action-objects correctly recalled, with four levels of the demonstration condition (no demo, object-only demo, action-only demo, full demo). This revealed a significant effect of condition, *F*(3,69) = 12.42, *MSE* = .113, *p* < .001, *η*^*2*^*p* = .35, *BF*_*10*_ = 11719. Further comparisons (Bonferroni-Holm corrected, *p* < .05) revealed significantly lower accuracy in the no-demo condition, relative to the action-only demo (*d* = .82, *BF*_*10*_ = 59.27), but not compared to the object-only demo (*d* = .39, *BF*_*10*_ = 1.00). The single feature (action or object) demo conditions did not differ (*d* = .14, *BF*_*10*_ = .263). Finally, the full-demo condition produced significantly higher accuracy than all other conditions (no demo, *d* = .96, *BF*_*10*_ = 276.45; action only, *d* = .70, *BF*_*10*_ = 17.07; object only, *d* = .83, *BF*^*10*^ = 68.63).

#### Actions

There was a significant effect of condition on verbal recall of actions, *F*(3,69) = 16.71, *MSE* = .14, *p* < .001, *η*^*2*^*p* = .42, *BF*_*10*_ = 462106. Further comparisons (Bonferroni-Holm corrected, *p* < .05) revealed significantly higher accuracy in the action-demo condition, compared to the no-demo condition (*d* = 1.28, *BF*_*10*_ = 8982), and compared to the object-demo condition (*d* = .93, *BF*_*10*_ = 208.75). Full demo also produced significantly higher accuracy compared to no demo, *d* = .98, *BF*_*10*_ = 343.42 and object demo, *d* = 1.03, *BF*_*10*_ = 593.71, but not compared to action demo, *d* = .06, *BF*_*10*_ = .22). There was no difference between object demo and no demo (*d* = .15, *BF*_*10*_ = .275).

#### Objects

There was a significant effect of condition on verbal recall of objects, *F*(3,69) = 16.63, *MSE* = .19, *p* < .001, *η*^*2*^*p* = .42, *BF*_*10*_ = 596919. Further comparisons (Bonferroni-Holm corrected, *p* < .05) revealed significantly higher accuracy in the object-demo condition compared to the no-demo condition (*d* = .87, *BF*_*10*_ = 104.84), and compared to the action-demo condition (*d* = .83, *BF*_*10*_ = 67.41). The full-demo condition also produced significantly higher accuracy than no demo, *d* = 1.00, *BF*_*10*_ = 417.97 and action demo, *d* = 1.15, *BF*_*10*_ = 2281, but not when compared to object demo, *d* = .13, *BF*_*10*_ = .256). There was no difference between action demo and no demo (*d* = .00, *BF*_*10*_ = .22).

### Discussion

This experiment replicated the beneficial effect of full demonstration on verbal recall of action-object pairs, when compared to a no-demonstration condition, as shown in previous FI studies (Waterman et al., 2017; Yang, Allen, & Gathercole, 2016a; Yang et al., 2017). Consistent with the results from Experiment 1, the current study showed that full demonstration improved verbal recall of actions. In contrast to Experiment 1, this study also found a beneficial effect of full demonstration on verbal recall of objects. This supports the idea that when objects are present at the encoding stage (as in Experiment 1), this leaves little scope for further improvement via reinforcing object information through demonstration. When the physical objects are removed from the procedure (as in Experiment 2), demonstration at encoding provides additional information that serves to boost subsequent recall. Indeed, comparing the mean object recall accuracy for the verbal-only conditions across Experiment 1 and Experiment 2 reveals a reduction in performance (from 78% correct to 62% correct), further supporting the idea that the availability of objects at encoding boosts object recall without the need for any additional cues provided by demonstration.

Experiment 2 also showed, for the first time, that full demonstration is also superior to partial demonstration, of either action or object, at least when considering the primary outcome variable of action-object pairs correctly recalled. Meanwhile, demonstration of a single feature benefits recall only for that feature, and not for the wider pairing in which it is embedded. This contradicts the conclusion that could have been drawn from Experiment 1 alone, that demonstration primarily serves to enhance encoding of action-related aspects of a visuo-motor sequence.

In conclusion, Experiment 2 provided further support for the positive effect of demonstration on verbal recall. It also showed, in contrast to Experiment 1, that demonstration can positively affect verbal recall of objects, in the same way as it does verbal recall of actions, when the availability of object/action information is made equivalent at the encoding stage. Finally, it provides novel insights into the effect of partial demonstration of individual features on different aspects of verbal recall within the FI paradigm.

## General discussion

Across two experiments, either without (Experiment 1) or with (Experiment 2) accompanying verbal presentation, visual demonstration consistently facilitated verbal recall of action-object pairs. Demonstration also improved memory for individual features when those features were not otherwise present in the environment (i.e. action in Experiment 1; either feature in Experiment 2). Furthermore, the interaction observed between demonstration and enactment in Experiment 1 would suggest overlap in how these encoding-based manipulations influence underlying cognitive processes. Finally, Experiment 2 decomposed demonstration into different levels of information and found that partial demonstration was not sufficient to boost recall of the object-action pairing. Instead it only boosted recall of the relevant individual feature.

Our results are consistent with the proposal that demonstration is likely to aid recall performance through provision of visual and spatial information concerning the actions to be performed and the objects on which these will be enacted. Indeed, it is well established that the addition or generation of visual information can benefit verbal memory (e.g. dual-coding hypothesis; Paivio, 1991). Some researchers have suggested that language comprehension can automatically activate motor circuits (Pulvermüller, 2005; Pulvermüller & Fadiga, 2010) but the present study would suggest that this is not as effective as physical enactment or demonstration. Instead we would suggest that, in the case of working memory, recall following verbal-only presentation relies primarily on phonological sequences that are only loosely chunked according to syntactic structure (e.g. Allen, Hitch, & Baddeley, 2018), which are more likely to be lost during encoding, storage, or retrieval.

Further insights concerning the mechanisms underlying demonstration effects can be derived from the interaction with enactment observed in Experiment 1, which suggests some representational overlap. Self-enactment is likely to increase engagement with visuospatial processing, with motor coding also playing a role (e.g. Allen & Waterman, 2015; Jaroslawska et al., 2018), but previous research by Waterman et al. (2017) would also suggest that this more active form of engagement may also generate competing, counter-productive, attentional demands. Meanwhile, in the case of demonstration, motoric representations may be automatically recruited by perceptual systems when observing another individual carrying out actions (Hickok & Poeppel, 2004; see also Rizzolatti & Luppino, 2001). In sum, both demonstration and self-enactment recruit additional, and likely highly similar, forms of coding that supplement the verbal codes. Given that the to-be-recalled instruction sequences are relatively long (four- and five-pair sequences equate to 12–15 words, respectively, including function words) this is likely beyond the capacity of phonological short-term memory. This further supports the idea that additional, or alternative, forms of coding within working memory are useful to supplement performance, in line with Logie’s (2011) description of a working memory system as a collection of cognitive functions that can be flexibly deployed in different ways, depending on the task.

A consistently emerging principle in the current study, and in the broader working memory literature on FI, is that changes in the task and materials between conditions are more likely to influence performance when they compensate for absence of information. Combining similar types of information does not have additive or multiplicative effects, and recall does not seem to benefit from redundancy. Instead, performance improves when changes in the methodology help ‘fill in’ what is missing from other conditions. It may prove useful to further test this general principle in future research, and to consider this point when applying implications from FI work to educational and learning contexts.

Experiment 2 indicated that when verbal presentation was accompanied by demonstration of one of the components of each action-object pairing representations were formed that benefitted recall for this component. Thus targeted demonstration of a feature (either action or object) can facilitate its recall. However, this improvement was limited to the demonstrated feature and did not appear to spread beyond this to any form of functional unit also containing accompanying features. Instead facilitation of memory for the complete action-object pair is only observed when the full pairing is demonstrated (or enacted). This would indicate that the instruction sequences used in the present study are maintained as loosely associated components rather than closely integrated units or chunks. The action-object pairings used in this study were deliberately arbitrarily constructed in order to focus on working memory function, with no pre-existing associations between these elements and a repeated use of re-combinations of these pairings from trial to trial (see also Allen & Waterman, 2015). Speculatively, the effects of partial demonstration of individual features might be more likely to generalize to other features within the broader to-be-remembered unit when using more familiar materials, with pre-existing associations between these elements. Such a finding would be in line with the notion that the functional units of representation in working memory can dynamically shift with changes in task, material and participant ‘repertoire’ (Macken, Taylor, & Jones, 2015).

Finally, we have already noted how a large body of work in the LTM domain has identified beneficial effects of enactment and observation on memory for actions and objects. While the underlying mechanisms driving these effects may have some parallels with those explored in the current exploration of working memory, it is not necessarily appropriate to draw direct conclusions across these distinct domains. Nevertheless, it may prove profitable for future investigations of the ability to retain and follow instructions in working memory to examine whether some of the same factors noted to be influential in LTM also apply in working memory. For example, work in the LTM domain has indicated that enactment and demonstration differentially impact on item and relational memory (e.g. Schult et al., 2014; Steffens, 2007), while the specific experimental design that is implemented (i.e. within- vs. between-subjects) appears to influence the magnitude of enactment effects that are observed (e.g. Steffens et al., 2015). It is not yet known whether such factors also apply to working memory. Similarly, there has been debate in the LTM literature concerning whether enactment effects represent activation of motor coding or integration of different episodic details including motor, visuo-spatial, and semantic information (e.g. Badinlou et al., 2018; Engelkamp, 1998; Kormi-Nouri, 2000). For example, Kormi-Nouri (2000) observed self-enactment benefits on a LTM task in sighted, blindfolded and blind participants, of a magnitude that somewhat contrasts with the relatively small effects observed in the present study. It may be valuable for subsequent research to explore whether distinct or overlapping forms of representational coding might contribute to encoding, retention, and retrieval of action-object information across the short- and long-term. Any such exploration would need to control for methodological differences as much as possible; variations in materials (e.g. geometric shapes vs. real objects), environmental support (e.g. the presence or absence of objects during encoding and retrieval), and experimental design (within- vs. between-subject manipulations), among other factors may also contribute to the emergence of distinct patterns of performance, beyond any theoretically derived effects.

Overall, the current study illustrates how enactment and demonstration can benefit verbal recall of instruction sequences. This may involve the co-opting of systems responsible for initially processing this information, in the service of organization and ongoing maintenance (e.g. Macken et al., 2015) and/or storage in specialized visuospatial and motor subsystems within working memory (Jaroslawska et al., 2018; see also Logie, 1995). When experimental conditions promote the engagement of multiple (verbal, visuospatial, motoric) representational codes these may then be drawn together in a consciously accessible, modality-general form (e.g. a focus of attention, Cowan, 2012, or episodic buffer, Baddeley, 2000, 2012) to enhance one’s ability to follow instructions.

## Electronic supplementary material

ESM 1 (CSV 6 kb)

ESM 2 (CSV 3 kb)

ESM 3 (TXT 3 kb)
